# Physical activity intervention promotes working memory and motor competence in preschool children

**DOI:** 10.3389/fpubh.2022.984887

**Published:** 2022-09-26

**Authors:** Jing-Yi Zhang, Qi-Qi Shen, Dong-Ling Wang, Jin-Mei Hou, Tong Xia, Shou Qiu, Xiao-Ye Wang, Si-Bo Zhou, Wen-Wen Yang, Si-Yu Heng, Can-Can Lu, Lei Cui, Heng-Chan Yin

**Affiliations:** ^1^College of P.E. and Sports, Beijing Normal University, Beijing, China; ^2^Branch School of Mingguang, Beijing Normal University Kindergarten, Beijing, China

**Keywords:** physical activity, exercise intervention, working memory, motor competence, preschool children

## Abstract

**Objective:**

This study investigated the effects of 12 weeks of specifically designed physical activity intervention on working memory and motor competence in preschool children and explored the correlation between working memory changes and motor competence changes by the intervention.

**Methods:**

Four classes of preschool children were grouped into an intervention group and a control group. Children in the intervention group received a 12-week physical activity intervention, while children in the control group followed their daily routine as usual. Before and after the intervention period, children were assessed with the 1-back task and Movement Assessment Battery for Children, second edition (MABC-2) to measure their working memory and motor competence, respectively.

**Results:**

Regarding working memory, the accuracy on the 1-back task increased significantly in the intervention group relative to the control group. The intervention group demonstrated a greater decrease in response time from pre- to posttest than the control group, but the difference was not statistically significant. Regarding motor competence, children's manual dexterity, aiming and catching and total score increased significantly in the intervention group relative to the control group, while no significant difference in static and dynamic balance was observed between the two groups. Furthermore, the correlation results showed that changes in the efficacy and efficiency of working memory were positively related to changes in static and dynamic balance and the total score on the MABC-2.

**Conclusion:**

These findings demonstrated that 12 weeks of specifically designed physical activity intervention could improve preschool children's efficacy of working memory as well as manual dexterity, aiming and catching and global motor competence. The improvement in the efficacy and efficiency of working memory was positively related to the improvement in static and dynamic balance and global motor competence.

## Introduction

Working memory, as one of the key components of executive function, refers to the collection of cognitive processes that temporarily retain information in an accessible state, which are suitable for carrying out any mental task (1–4). Working memory is essential for a wide range of cognitive abilities, such as understanding written or spoken language, reasoning, and making plans and decisions (5), particularly those that require dealing with interference, conflict, or distraction (6), and it predicts important cognitive and academic outcomes in children. Working memory deficits, on the other hand, have negative repercussions. Preschool children are in a critical period of working memory development. Improving their working memory has become a concerning problem. Meta-analytic studies have found that working memory can be trained (7–9). Computer-based training, educational programs, and physical activities have all been offered as ways to foster children's working memory (10–14). Physical activity interventions that are consistently challenging, playful, enjoyable, and cognitively enriched appear to have a more positive impact on children's working memory (13, 15).

Motor competence is defined as the ability to perform a wide range of motor skills that require motor coordination and control and that primarily include fundamental locomotor (e.g., jumping and running) and object-control skills (e.g., catching and throwing) (16). These competences are continuously developed in the early years and are further refined into context- and sport-specific skills (17). Good motor competence is seen to be vital for children's physical, social, and psychological development (18) as well as being the foundation for an active lifestyle, whereas poor motor competence is linked to less involvement in sports activities (19). The initial years of a person's life are especially important for developing their motor competence, which does not develop naturally or automatically over time but is learned, practiced, and developed (20, 21). According to this view, intervention programs with structured learning environments and specific purposes are critical for motor competence development in children (22–24). Placing children in environments with sensory, cognitive and social stimulation could inspire their exploration, curiosity and enjoyment in as early as preschool, which could lead to optimal development (25).

Collectively, early childhood is a period when children's physical, motor and cognitive abilities are especially rapidly developed (26). Carrying out well directed and planned Physical Education programs is essential for children's physical, social, and emotional wellbeing, and for increasing adherence to physical exercise (27). Studies have mostly explored the influences of physical activity on either motor competence or working memory independently; however, evidence on the effects of specifically designed interventions on both motor competence and working memory is still lacking. Furthermore, the relationship between motor competence and working memory is still unclear.

As a result, the goal of this study was to determine whether a specifically designed physical activity intervention may help preschool children improve their working memory and motor competence. There were two hypotheses of this study: (1) a long-term physical activity intervention may cause changes in working memory and motor competence, and (2) working memory changes caused by physical activity intervention are associated with motor competence changes.

## Materials and methods

### Participants and study design

Four classes of children from a kindergarten school located in Beijing were invited to participate in the study. Among a total of 126 preschool children aged 4 to 5 years, 17 were excluded for the following reasons: (a) their parents did not sign a consent form (*N* = 2); (b) they did not complete related tests for various reasons (i.e., developmental delays or not attending school during the testing period) (*N* = 8); and (c) their attendance rates fell below 70% (*N* = 7). The final sample consisted of 109 participants. The guardians of all the participants were fully informed of the experimental contents and signed written informed consent.

Due to the convenience and not disturbing the kindergarten's schedule, the experiment was organized by class. Four classes (Class 1–4) were divided into two groups based on children's physical activity levels (a survey from their guardians) and pretest scores to ensure homogeneity, with every two classes being combined as a whole. As a result, Class 1 and Class 3 combined to form a single group, and Class 2 and Class 4 combined to form the other group. All dependent variables of the two groups remained no significant difference on pre-test. Then, the two groups were randomly designated the intervention group (*N* = 57) and control group (*N* = 52). No significant differences in sex, age or dominant hand were observed between the two groups. Detailed sample characteristics are shown in Table 1.

**Table 1:** Sample characteristics.

	**Intervention group (*n* = 57)**	**Control group (*n* = 52)**	***t***	***p***
Gender (male/female)	28/29	26/26	–	–
Age (M ± SD)	4.51 ± 0.31	4.53 ± 0.28	0.2570	0.798
Dominant hand (right/left)	57/0	51/1	–	–

The experiment used a 2 (group: intervention group, control group) × 2 (time: pre, post) mixed factorial design with time as a within-subjects factor and group as a between-subjects factor. The 1-back task and Movement Assessment Battery for Children, second edition (MABC-2) were used to measure children's working memory and motor competence, respectively, both before and after the intervention.

### Physical activity intervention procedures

During the 12-week intervention period, children in the intervention group received physical activity interventions (three 40-min sessions weekly), while children in the control group engaged in regular activities as usual. For most of the time, the intervention was conducted on Tuesday, Wednesday, and Friday. We conducted the intervention on Tuesday instead of Monday because the kindergarten pre-arranged their own activity on Monday. And as we tried to avoid four consecutive days' lack of intervention during a week, we did not arrange the intervention on three consecutive days. However, in some cases (i.e., the intervention day is on a public holiday), the arrangement would be adjusted, but three intervention days a week need to be covered. The interventions were carried out at the outdoor playground at the school; however, in some cases of severe weather, they were carried out in a multipurpose room with ample space. All the interventions were conducted in the morning, and the instructors are sport psychology students with qualified teacher certifications.

The activities included two types of games. Type 1 games mainly focused on motor learning with the purpose of allowing children to acquire fundamental movement skills, including locomotor (e.g., jumping, running, hopping, leaping, sliding, and galloping) and object control skills (e.g., catching, throwing, dribbling, rolling, kicking, and striking), to set the foundation for more challenging and complex games soon afterward. Type 2 games were based on type 1 games but incorporated more cognitive rules that were specifically designed to foster children's cognitive abilities with the purpose of promoting working memory and motor competence jointly. Since cognitive abilities are distinguishing but also integrating, and they influence and are influenced by each other, cognitive rules in the games included but were not limited to the core elements of working memory. A summary of the main games used is featured in Table 2. All games were designed for the children's enjoyment, enthusiasm, and engagement.

**Table 2:** Summary of six main type 2 games in the physical activity intervention.

**Game**	**Description**	**Cognition involvement**
Delayed Imitation (Same/ Opposite direction)	The teacher and children stand at a fixed sign point. When the music starts, the teacher engages in a movement (i.e., jump to the left of the sign point with hands on each side of his or her waist). The children are asked to observe the teacher's movement, and once the teacher is finished, they are required to imitate the teacher by performing the same movement in the same direction. As the children become familiar with the rules of the game, the teacher gradually increases the level of difficulty: (1) one simple movement changes to a sequence of complex movements (i.e., movements that require hand and foot coordination), and (2) the children are asked to imitate the teacher's movement, but in the opposite direction.	Remember the sequence of movements, update the sequence of movements, translate instructions into action plans, inhibit the urge to make habitual movements, adapt to rule changes, etc.
Delivering watermelons	The children stand in a line, keeping their feet the same distance apart, at shoulder width. Each child must maintain a certain distance from the next. The child at the front of the line holds a large watermelon toy in his/her hands and passes it backwards from the top of his/her head (or between two legs) to the child standing behind him/her. After the pass, the child quickly runs to the end of the line and waits for the next pass. Then the second child begins to pass it to the next one, just as the first child did, and so on. Once the children are familiar with the routine of the game, more rules are added or changed: (1) the children need to pass the watermelon in a specified order as the teacher guides their actions. For example, the children are asked to pass the watermelon “one top, one bottom” (i.e., over their head, then through their legs), and then alternating, with the first child passing it over his or her head, and the next one passing it between his or her legs. 2)The children are asked to perform a similar order of operations, but in a longer sequence, such as “top-bottom-left-right” or “bottom-bottom-left-right.” 3) There is no pre-determined order the children need to obey, but they need to pass the watermelon in a direction different from previous child. 4) Each child is given an animal role, with every animal corresponding to a specific direction for passing. As the children become familiar with the animal-direction combination, the combination of animals is changed.	Remember the sequence of passing directions; remember the pre-determined direction that the teacher provides, inhibit the urge to rely on habitual directions of delivery, apply the pre-determined order to their actions, adapt to rule changes, etc.
Little basketball protector	The children are asked to dribble a basketball and protect it from being ‘snatched' by the teacher while energetic music plays in the background. The play area where this game takes place should include different colored ‘safety zones,' which correspond to different movements (e.g., children in the green safety zone need to stand on one leg). When the music stops, the children are to run quickly into a safety zone and perform the corresponding movement for that zone. As the children became familiar with the basic rules of the game, more complex rules are added; for example, the children are asked (not) to go to the same colored safety zones twice consecutively. Once children become familiar with the color-movement combination, the game begins anew.	Remember the color of the ‘safety zone' for each session, remember the correspondence between colors and movements, apply game rules to actions, adapt to rule changes, etc.
Quickly positioned	Each body part corresponds to a kind of vehicle (e.g., forehead corresponds to a plane, shoulders correspond to a train, knees correspond to a ship). When the teacher says a vehicle name, the children are to quickly put their hands on the corresponding body part. It is also possible to use the vehicle to correspond to different movements. When the children hear a vehicle name, for instance, they are to perform the corresponding movement (e.g., the teacher says ‘plane,' so the children should hop on one foot). Once the children are familiar with the basic rules of the game, more complex rules will be added: (1) the teacher says two or three vehicles in a row and asks the children to touch the corresponding body parts in the order in which they hear them; or (2) the teacher says a sequence of vehicles and asks the children to touch the body part that corresponds to the first/second/third vehicle they hear. Once the children are familiar with the body-vehicle combination, a new match will be set.	Remember the relationship between body parts and vehicles, remember the vehicle order that the teacher specifies, update the order for each session, adapt to rule changes, etc.
Animal story	Each child takes on the role of an animal. The teacher says three animals in a row (e.g., tiger, lion, and rabbit), and the ‘animal' called will be asked to carry food from a storage area to a ‘canteen' (a designated place). The children need to remember their animal role and the order in which they are called. The order the children are called represents the number of food items they need to deliver. For example, if the rabbit is the third one called, then the children assuming the rabbit role need to pick three food items (e.g., apples) to bring to the ‘canteen.' As the children become familiar with the rules, the difficulty will increase: (1) each animal needs to adopt a different movement (e.g., a tiger needs to hop, a lion needs to skip, and a rabbit needs to jump); (2) when the children need to pick more than one kind of food, the food should be the same/different. Once the children become familiar with their role, their roles will be changed.	Remember their own animal role, remember the order they are picked, update the order for each session, apply the rules to their actions, adapt to rule changes, etc.
Colored flags	The children run on a playground while background music plays. When the music stops, the children are asked to perform different movement-based tasks according to the color of the flag in the teacher's hand (i.e., a red flag signals to turn around, a green flag to jump, a yellow flag to stand on one leg). After the children are familiar with the rules: (1) the color-movement combination will be changed; (2) different rhythms of background music will be added and matched to different movement-related tasks.	Remember the combination of color and movement, apply the rules to their actions, adapt to the rhythm of the music, adapt to rule changes, etc.

The 12 weeks of intervention were divided into 3 phases. In the first 4 weeks (Phase 1), type 1 games were introduced because simple, less difficult motor games were assumed to be sufficiently challenging for the children since many of the fundamental skills being introduced were not yet familiar to them. In the middle 4 weeks (Phase 2), type 2 games were included, with some type 1 games still remaining because after 4 weeks of practice in phase 1, some motor skills had become automated, and more difficult games were needed. In the last 4 weeks (Phase 3), there remained a combination of type 1 and type 2 games, but the difficulty level continued to rise to increase the chance of reaching the children's optimal challenge level (28, 29).

For each 40-min intervention session, a 5-min warm-up, 30-min formal activities, and a 5-min cool-down were needed. During the warm-up and cool-down, children were mainly led to stretch their whole body with low-intensity activities for the sake of fully preparing for the intervention (warm-up) and relaxing their body (cool-down). Furthermore, the practice of deep breathing was also included in the cool-down part to help them calm down and prepare for the next routine of kindergarten. The 30-min formal activities were pre-arranged games with higher intensity. Throughout the session, the children's heart rates (HR) were monitored by polar watch or a manual check of their radial arteries to ensure moderate intensity physical activity (60% to 69% HRmax).

### Data acquisition

#### Working memory assessment

A modified 1-back task was applied to measure the participants' working memory before and after the intervention. The 1-back task is taken from the *n*-back paradigm, which is widely used as a measure of working memory. In the *n*-back task, subjects identify over consecutive trials whether the current stimulus matches a stimulus presented *n* trials before (30). Based on preschoolers' cognitive abilities, we adopted a computerized pictorial 1-back task to test their working memory, presented on E-prime 1.0.

The target stimuli were composed of 5 different geometric shapes of different colors to suit the children's cognitive level. Before the experiment task, the experimenter fully explained the rules of the game with the help of the geometric pictures printed on the white paper. After the children reported that they fully understood the rules, they began to work on the experimental task. The task involved practice and formal sessions with 12 and 48 trials, respectively. For each trial, the target stimulus was presented on a screen for 3000 ms, which was followed by a white blank screen presented for 1000 ms. Children were required to make judgments as accurately and quickly as possible by pressing a button. All trials were presented in randomized order, and for each stimulus, the children were asked to determine whether the picture presented on the screen was the same as the one that had been shown previously. Response accuracy (ACC) and reaction time (RT) during formal sessions were collected to assess working memory performance. The higher the ACC, the higher the efficiency, in contrast, the lower the RT, the higher the efficacy.

#### Motor competence assessment

The MABC-2 was used for each participant to measure their motor competence before and after the intervention period. It is a revision of the MABC, one of the most broadly used assessment tools by psychologists, physiotherapists, and educational professionals (31), which assesses children's motor competence on eight tasks for three age bands (3~6 years, 7~10 years, and 11~16 years) (32). These tasks are categorized to assess three motor skills: manual dexterity, aiming and catching, and static and dynamic balance. Their scores are combined into a total score as a general indicator of global motor competence.

During the test, for each test item, children were allowed to make two attempts, with their best attempt used as the score. An F (failure) score was given when a child failed to complete a task or and an R (refusal) score was given when a child refused to perform a task.

Raw item scores were converted into age-adjusted standard scores using the officially licensed system (Standardized Assessment System for Children's Motor Coordination, Chinese version; https://movementabc.online/yjy/#/home). In the system, F and R scores would automatically convert into 1, which is the minimum standard score. Three standard scores that assess manual dexterity, aiming and catching, and static and dynamic balance separately, and a total standard score that assesses global motor competence were used as the dependent measures.

### Statistical analysis

Analyses were conducted using SPSS 26.0 (International Business Machines Corp., NY, USA).

For all of the variables, independent-sample *t* tests were used to assess whether participants in the two groups differed on baseline scores. A repeated-measures ANOVA was then applied to explore task performance changes in the intervention group and control group from pre- to postintervention. A simple effect test was subsequently performed when the interaction effect was significant.

Finally, a Pearson correlation analysis was used to estimate the relationship between changes in working memory and changes in motor competence with physical activity intervention for children in the intervention group.

For all statistical results, *p* ≤ 0.05 (^*^) was considered significant, *p* ≤ 0.01 (^**^) was considered highly significant, *p* ≤ 0.001 (^***^) was considered strongly significant and *p* > 0.05 was considered non-significant.

## Results

### Baseline characteristics

An independent-sample *t* test of the pretest scores showed no significant differences between the two groups with respect to the 1-back task (ACC) (*t* = −0.616*, p* = 0.539), 1-back task (RT) (*t* = −0.070, *p* = 0.944), manual dexterity (*t* = −0.072*, p* = 0.943), aiming and catching (*t* = −0.749, *p* = 0.455), static and dynamic balance (*t* = 1.457, *p* = 0.148), and total score (*t* = 0.760, *p* = 0.449), indicating group homogeneity before the physical activity intervention was implemented (Table 3).

**Table 3:** Baseline characteristics of participants.

	**Intervention group (M ±SD)**	**Control group (M ±SD)**	***t***	***p***
**Working memory**				
1-back task (ACC)	73.830 ± 15.304	71.895 ± 17.462	−0.616	0.539
1-back task (RT)	1769.385 ± 301.854	1765.070 ± 337.637	−0.070	0.944
**Motor competence**				
Manual dexterity	8.67 ± 2.190	8.63 ± 2.458	−0.072	0.943
Aiming and catching	10.09 ± 2.355	9.71 ± 2.879	−0.749	0.455
Static and dynamic balance	9.88 ± 3.268	10.75 ± 2.956	1.457	0.148
Total score	9.18 ± 2.354	9.54 ± 2.631	0.760	0.449

### Working memory results

**Figure 1:**
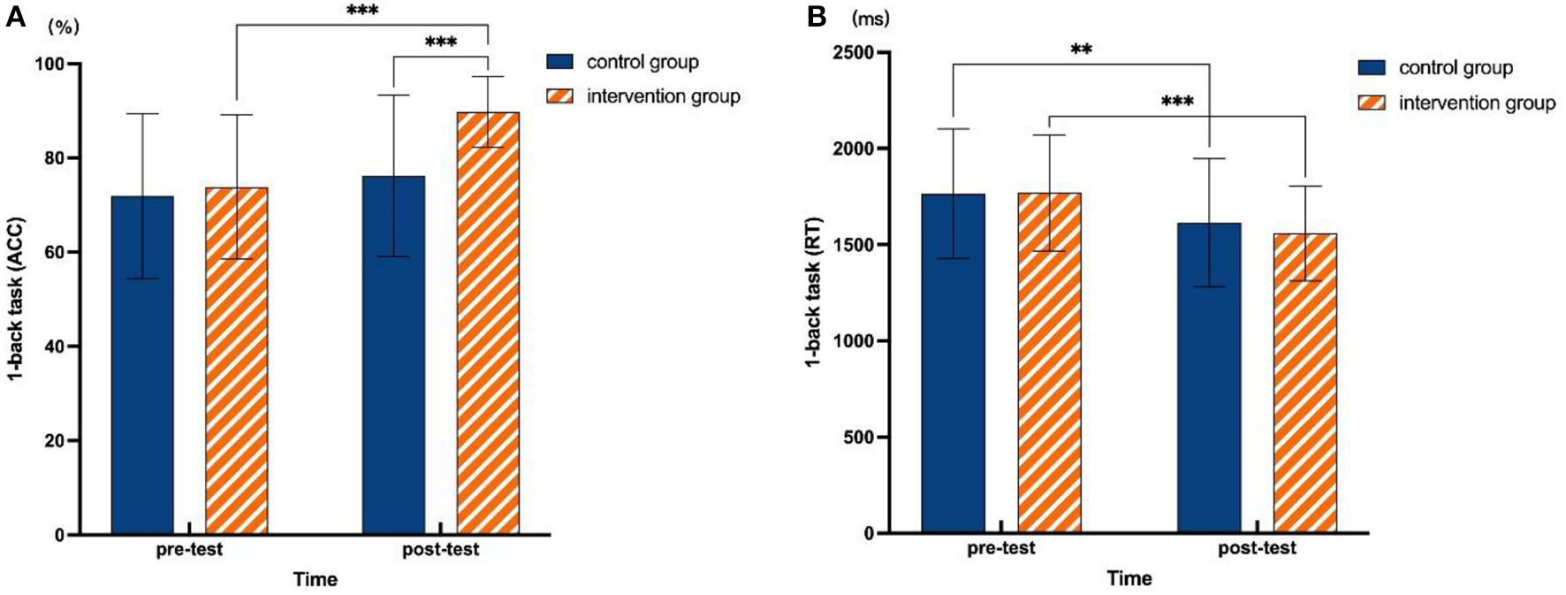
Changes in 1-back task performance. **(A)** Simple effect analysis results in 1-back task (ACC). **(B)** Simple effect analysis results in 1-back task (RT). **p ≤ 0.01, ***p ≤ 0.001.

For the ACC of the 1-back task, the results revealed a significant main effect of time [*F*_(1, 107)_ = 38.654, *p* < 0.001, $\eta_{p}^{2}$ = 0.265], main effect of the group [*F*_(1, 107)_ = 11.171, *p* = 0.001, $\eta_{p}^{2}$ = 0.095], and time × group interaction [*F*_(1, 107)_ = 12.810, *p* = 0.001, $\eta_{p}^{2}$ = 0.107] (see Table 4). Further results of simple effect analysis showed a significant difference between pre- and posttest results for the intervention group with higher ACC found for posttest than pretest results [*F*_(1, 107)_ = 50.291, *p* < 0.001, $\eta_{p}^{2}$ = 0.320] but no significant difference observed between pre- and posttest results in the control group [*F*_(1, 107)_ = 3.327, *p* = 0.071, $\eta_{p}^{2}$ = 0.030]. Moreover, there was no significant difference between the intervention group and control group for the pretest [*F*_(1, 107)_ = 0.380, *p* = 0.539, $\eta_{p}^{2}$ = 0.004], but there was a significant difference for the posttest [*F*_(1, 107)_= 29.613, *p* < 0.001, $\eta_{p}^{2}$ = 0.217], with the ACC of the intervention group being significantly higher than that of the control group (see Figure 1).

**Table 4:** 1-back task performance.

	**Source**	***p***	** $\eta_{p}^{2}$ **
1-back task (ACC)	Time	< 0.001***	0.265
	Group	0.001***	0.095
	Time*group	0.001***	0.107
1-back task (RT)	Time	< 0.001***	0.213
	Group	0.594	0.003
	Time*group	0.375	0.007

^***^p ≤ 0.001.

Regarding RT of the 1-back task, there was a significant main effect of time [*F*_(1, 107)_ = 28.971, *p* < 0.001, $\eta_{p}^{2}$ = 0.213], indicating that the RT changed significantly between pre- and posttests (Table 4). Further analysis showed that the RT decreased significantly for the posttest relative to the pretest, both in the intervention group [*F*_(1, 107)_ = 9.646, *p* < 0.001, $\eta_{p}^{2}$ = 0.162] and control group [*F*_(1, 107)_ = 20.623, *p* = 0.002, $\eta_{p}^{2}$ = 0.083], but no significant difference between the two groups was observed [*F*_(1, 107)_ **=** 0.993, *p* = 0.321, $\eta_{p}^{2}$ = 0.009] for the posttest (Figure 1). Independent sample *t* test results showed no significant difference between the intervention group and control group in terms of changes in RT (posttest – pretest) (*t* = 0.891, *p* = 0.375).

### Motor competence results

**Figure 2:**
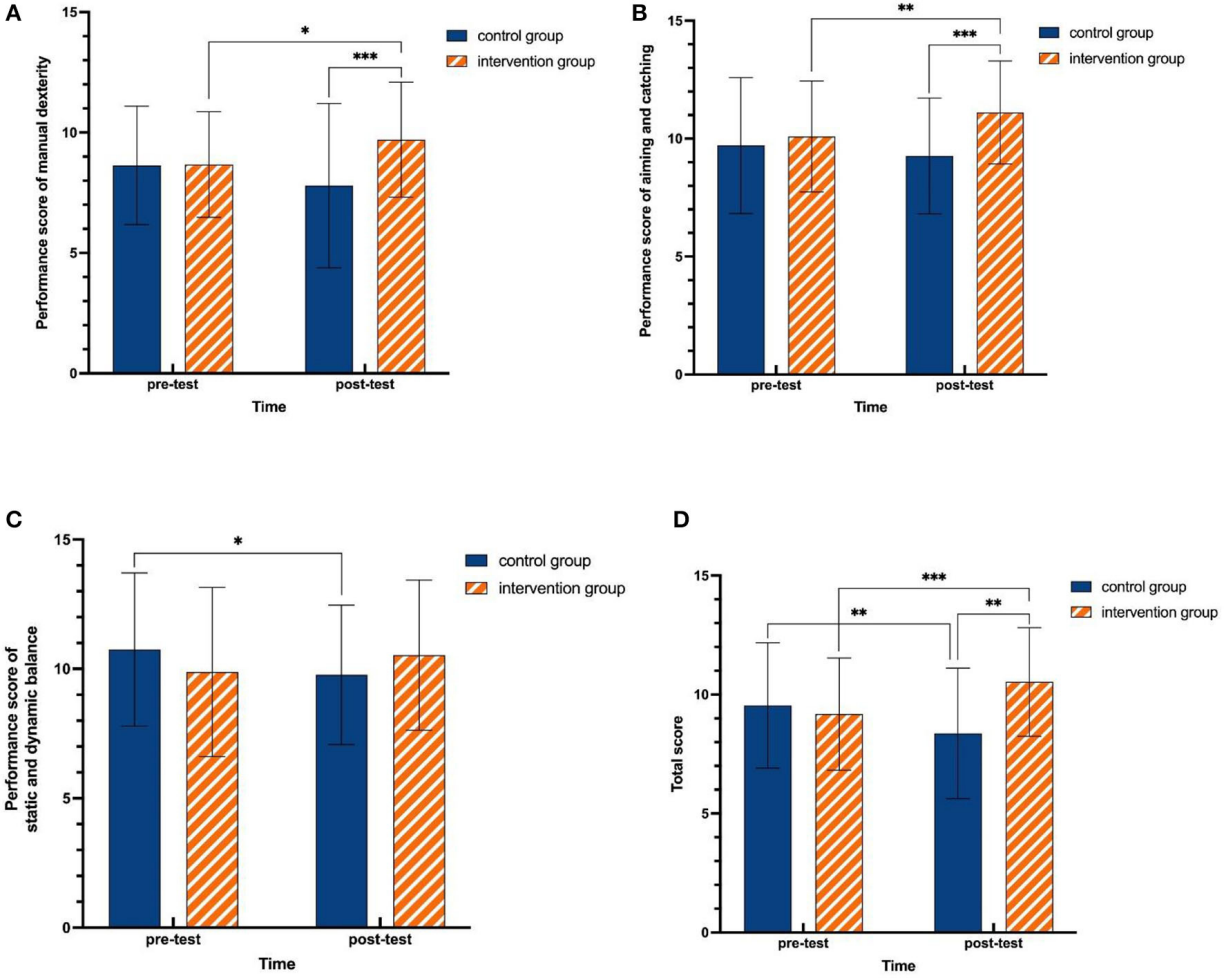
Changes in MABC-2 performance. **(A)** Simple effect analysis results in manual dexterity. **(B)** Simple effect analysis results in aiming and catching. **(C)** Simple effect analysis results in static and dynamic balance. **(D)** Simple effect analysis results in total score. *p ≤ 0.05, **p ≤ 0.01, ***p ≤ 0.001.

For manual dexterity, the results revealed a significant main effect of group [*F*_(1, 107)_ = 5.777, *p* = 0.018, $\eta_{p}^{2}$ = 0.051] and a time × group interaction [*F*_(1, 107)_ = 9.564, *p* = 0.003, $\eta_{p}^{2}$ = 0.082], and no significant main effect of time was found [*F*_(1, 107)_ = 0.096, *p* = 0.757, $\eta_{p}^{2}$ = 0.001] (Table 5). Further results of simple effect analysis showed a significant difference between pre- and posttest results for the intervention group [*F*_(1, 107)_ = 6.069, *p* = 0.015, $\eta_{p}^{2}$ = 0.054] with higher posttest than pretest scores, but no significant difference was observed between pre- and posttest results for the control group [*F*_(1, 107)_ = 3.700, *p* = 0.057, $\eta_{p}^{2}$ = 0.033]. Moreover, no significant difference between the intervention group and control group on the pretest [*F*_(1, 107)_ = 0.005, *p* = 0.943, $\eta_{p}^{2}$ < 0.001] was observed, but there was a significant difference for the posttest [*F*_(1, 107)_ = 11.646, *p* = 0.001, $\eta_{p}^{2}$ = 0.098], with scores of the intervention group being significantly higher than those of the control group (Figure 2).

**Table 5:** MABC-2 performance.

	**Source**	***p***	** $\eta_{p}^{2}$ **
Manual dexterity	Time	0.757	0.001
	Group	0.018*	0.051
	Time*group	0.003**	0.082
Aiming and catching	Time	0.281	0.011
	Group	0.006**	0.069
	Time*group	0.007**	0.066
Static and dynamic balance	Time	0.615	0.002
	Group	0.901	< 0.001
	Time*group	0.015*	0.054
Total score	Time	0.735	0.001
	Group	0.027*	0.045
	Time*group	< 0.001***	0.178

^*^p ≤ 0.05, ^**^p ≤ 0.01, ^***^p ≤ 0.001.

For aiming and catching, the results revealed a significant main effect of group [*F*_(1, 107)_ = 7.920, *p* = 0.006, $\eta_{p}^{2}$ = 0.069] and time × group interaction [*F*_(1, 107)_ = 7.564, *p* = 0.007, $\eta_{p}^{2}$ = 0.066], but no significant main effect of time was observed [*F*_(1, 107)_ = 1.174, *p* = 0.281, $\eta_{p}^{2}$ = 0.011] (Table 5). Further results of simple effect analysis showed a significant difference between pre- and posttest results for the intervention group [*F*_(1, 107)_ = 7.703, *p* = 0.007, $\eta_{p}^{2}$ = 0.067] with higher posttest than pretest scores, but no significant difference was observed between pre- and posttest scores in the control group [*F*_(1, 107)_ = 1.328, *p* = 0.252, $\eta_{p}^{2}$ = 0.012]. Moreover, there was no significant difference between the intervention group and control group for the pretest [*F*_(1, 107)_ = 0.562, *p* = 0.455, $\eta_{p}^{2}$ = 0.005], but there was a significant difference for the posttest [*F*_(1, 107)_ = 17.040, *p* < 0.001, $\eta_{p}^{2}$ = 0.137], with scores in the intervention group being significantly higher than those in the control group (Figure 2).

For static and dynamic balance, the results revealed a significant time × group interaction [*F*_(1, 107)_ = 6.135, *p* = 0.015, $\eta_{p}^{2}$ = 0.054] (Table 5). The time × group interaction effect was further analyzed using simple effect analysis. The results showed that for the intervention group, the posttest scores were much higher than the pretest scores, but the difference was not statistically significant [*F*_(1, 107)_ = 2.040, *p* = 0.156, $\eta_{p}^{2}$ = 0.019]; there was a significant decrease in posttest scores compared to pretest scores in the control group [*F*_(1, 107)_ = 4.248, *p* = 0.042, $\eta_{p}^{2}$ = 0.038]. Moreover, there was no significant difference between the two groups after the experiment [*F*_(1, 107)_ = 1.987, *p* = 0.162, $\eta_{p}^{2}$ = 0.018] (Figure 2).

In terms of total score, the results revealed a significant main effect of group [*F*_(1, 107)_ = 5.007, *p* = 0.027, $\eta_{p}^{2}$ = 0.045] and time × group interaction [*F*_(1, 107)_ = 23.157, *p* < 0.001, $\eta_{p}^{2}$ = 0.178], but no significant main effect of time was observed [*F*_(1, 107)_ = 0.115, *p* = 0.735, $\eta_{p}^{2}$ = 0.001] (Table 5). Further results of simple effect analysis showed a significant improvement for the posttest from the pretest in the intervention group [*F*_(1, 107)_ =13.905, *p* < 0.001, $\eta_{p}^{2}$ = 0.115], while there was a significant decrease from the posttest to the pretest [*F*_(1, 107)_ = 9.566, *p* = 0.003, $\eta_{p}^{2}$ = 0.082] in the control group. Moreover, there was no significant difference between the two groups for the pretest [*F*_(1, 107)_ = 0.578, *p* = 0.449, $\eta_{p}^{2}$ = 0.005], while a significant difference between them for the posttest was observed [*F*_(1, 107)_ = 20.091, *p* < 0.001, $\eta_{p}^{2}$ = 0.158], with scores in the intervention group being significantly higher than those in the control group (Figure 2).

### Correlations between working memory and motor performance changes

Correlation analysis results of changes in working memory and motor competence for children in the intervention group showed significant positive correlations between changes in the static and dynamic balance and 1-back (ACC) (*r* = 0.289, *p* = 0.029) and changes in the total score and 1-back (ACC) (*r* = 0.293, *p* = 0.027) (Table 6). Additionally, significant negative correlations were found between changes in the static and dynamic balance and 1-back (RT) (*r* = −0.419, *p* = 0.001) and changes in the total score and 1-back (RT) (*r* = −0.341, *p* = 0.009) (Table 6). The results indicated that the improvements of the efficacy (increase in ACC) and efficiency (decrease in RT) of working memory were positively related to the improvement of skills of static and dynamic balance and global motor competence. However, one thing should also be noticed that except for the moderate correlation between changes in static and dynamic balance and 1-back (RT), the other correlations are weak.

**Table 6:** Correlations between motor performance and working memory changes.

	**Changes of 1-back (ACC)**	**Changes of 1-back (RT)**
Changes in static and dynamic balance	*r* = 0.289, *p* = 0.029*	*r* = −0.419, *p* = 0.001***
Changes in total score	*r* = 0.293, *p* = 0.027*	*r* = −0.341, *p* = 0.009**

^*^p ≤ 0.05, ^**^p ≤ 0.01, ^***^p ≤ 0.001.

## Discussion

The goal of this study was to examine the effects of an physical activity intervention on promoting working memory and motor competence in preschool children. We found that physical activity intervention significantly increased the efficacy of working memory, manual dexterity, aiming and catching, and global motor competence. Furthermore, there was a significant positive correlation between improved working memory performance (both ACC and RT) and improved static and dynamic balance as well as global motor competence for the intervention group.

### Effects of the physical activity intervention on working memory

Our findings showed that a 12-week physical activity intervention specifically tailored to preschool children enhanced their working memory, which is consistent with previous research (33).

According to meta-analytical studies, a favorable impact on working memory was observed after an extended period of increased physical activity (34). Training studies have also evidenced a relationship between physical activity programs and improved working memory (33, 35, 36). In addition, among the various intensities of physical activity, moderate-intensity physical activity is considered to be the most effective at improving cognition (37, 38). In our study, the physical activity intervention lasted 12 weeks, with moderate intensity throughout, resulting in better working memory performance for children in the intervention group than in the control group.

Except for the positive influences of physical activity itself, cognitive elements engaged in the intervention were also an important contributor to improving participants' working memory. Previous studies have revealed that cognitively enriched programs have the potential to improve cognitive abilities than physical activities with less cognitive content. Mirko Schmidt et al. (28) compared the effects of a 6-week combined physical-cognitive intervention vs. a sedentary cognitive intervention and a control group and found that children from two cognitively engaged groups improved their working memory performance compared to children in the control group. A pilot study from Valentina Biino et al. (39) investigated the influences of cognitively enriched physical activity on motor skills and executive functions in preschool children and found that after a 12-week intervention, children who received the cognitively enriched physical activity intervention showed significant improvements in working memory and gross motor skills compared to those who were assigned to swimming and the control group. These studies suggested that physical activity intervention with cognitive engagement is a viable way to improve working memory at as early as preschool age. The games we utilized in our study incorporated rich cognitive elements into physical activity games that targeted promoting motor and working memory abilities jointly. For example, in a game called “Delayed Imitation”, teachers performed a sequence of specified movements in the first four beats, and children were asked to imitate these movements in the following four beats. The game required children to use a variety of cognitive abilities, including working memory, which helped them keep the sequence of movements in mind before imitating successively. With continuous practice of these games, the children's working memory improved.

Notably, in our study, the improvement in working memory in the physical activity intervention was mainly reflected in efficacy (ACC) rather than efficiency (RT). It may be that compared to RT, ACC is a more sensitive and reliable measure of working memory tasks in childhood (40, 41), which differs from tasks for young adults, where RTs are more reliable than accuracies under the same conditions (42, 43). Beyond the fact that the physical activity intervention did not significantly enhance the efficiency of working memory, we also found that compared to performance on the pretest, the efficiency of working memory for children in both groups was significantly enhanced with the posttest. Since previous studies have found that rapid improvements in working memory tasks occur during preschool and early school years (26, 44, 45), it is reasonable that regardless of whether an intervention was received, children's performance on RT for the 1-back task significantly improved.

### Effects of the physical activity intervention on motor competence

The results showed that in general, the 12-week PA intervention improved children's motor competence. This is consistent with previous reviews that indicated positive effects of preschool-based interventions on fundamental movement skill proficiency in early childhood (21, 46, 47), suggesting that specific interventions could positively affect children's motor competence. However, we also noticed that static and dynamic balance were not improved after the intervention. This may be due to the emergence of a ‘ceiling effects.' Ceiling effects occur when the scores of a relatively large proportion of a sample are in the upper range of the measurement scale (48, 49). There are four tasks used to assess static and dynamic balance, and the sum of these four tasks' standard scores is equal to the final standard score for static and dynamic balance. As we observed, a large proportion of children in the intervention group reached ‘ceiling effects' on the pre-test (the same as children in the control group), with about 47.4% and 71.9% of them receiving the highest score on the ‘walking heels raised' and ‘jumping over cord' tasks, respectively. This resulted in a relatively high score for static and dynamic balance on the pretest. Therefore, although the children's performance improved on the posttest, there was no significant difference identified. Since the proportion of children who reached the highest score on the two tasks is similar between the two groups, there was no significant difference both on pre-test and post-test for static and dynamic balance.

The first years of life are an essential period for developing motor competence. A series of studies showed that children who learned motor skills under specialist guidance showed greater growth in motor competence than children engaged in free play (50–52). This indicates that in addition to natural development and maturation, continuous interaction with a stimulating and supportive social and physical environment as well as professional instructions also benefit children's motor competence (46, 53, 54). The physical activity intervention used in our study is based on fundamental movement skills requiring motor coordination and control, providing many opportunities for participants to learn and practice under the guidance of professional instructors in attractive environments and settings to can maximize their development of motor competence.

The results also showed that for participants in the control group, there was no difference in manual dexterity, aiming and catching between the pretest and posttest. Because of the significant decrease in static and dynamic balance, the total score also decreased significantly. These results surprised us because it was expected that due to normal development and maturation, children's motor competence should increase even without specific intervention (55). Possible reasons for the decrease in static and dynamic balance may include a mis-estimation of the children's abilities as a result of ‘ceiling effects,' and bias in the process of converting raw scores to age-adjusted standard scores. Another possible reason that cannot be ignored may be that the pretest occurred at the beginning of a new semester after a long summer holiday, when children were expected to have engaged in more physical activity; therefore, they had many opportunities to acquire better motor competence. The posttest occurred at the end of the semester. The semester ran from summer to winter, and children's physical activity decreased during this period, so their performance on some of the tasks declined. This indicated that without adequate physical activity experience, children's motor competence may decrease.

### Correlations between working memory and motor performance

The correlation results showed that the improvement of working memory was positively related to the improvement of static and dynamic balance and global motor competence, indicating that the development of motor competence and the maturation of working memory are interrelated (56). From a neuroscientific point of view, their connection may be due to the fact that they share overlapping neural mechanisms and draw on common resources (57–61). Research has found that working memory and its neural circuitry follow a trajectory similar to that of motor competence (45), as the basal ganglia, prefrontal cortex and cerebellum are coactivated for top-down control of behavior in both complex cognitive and movement tasks (62, 63). Research from the behavioral sciences has also found that interventions aimed at promoting motor skills could also improve the performance of working memory (63–66), and the program of motor activities linked to executive functions significantly improved both motor competence and executive functions (67), indicating a positive relation between working memory and motor competence.

In this study, the games we used in the intervention involved the deep engagement of motor and cognitive abilities, which contributed to the joint improvement of motor competence and working memory. Type 1 games involved process of motor learning and control. When participating in these games, children managed new stimuli, meaning that to perform motor skills better, cognitive abilities such as working memory were activated (62). Type 2 games combined motor skills and cognitive rules to provide opportunities to practice motor competence and working memory at the same time. As the games became more difficult, children gained experience working with new stimuli and practicing complex motor skills, which led to the improvement of their motor competence and working memory.

## Conclusion

In summary, our study indicated that a 12-week specifically designed physical activity intervention could improve preschool children's working memory and motor competence. Additionally, the improvement in the efficiency and the efficacy of working memory was found to be positively related to the improvement in static and dynamic balance and global motor competence.

## Study limitations

The present results suggest an effective way of promoting preschool children's working memory and motor competence jointly. In addition to the contributions of this study, we must consider some limitations and thus view the results with caution. In our study, only two groups were set, so as to compare the effects of a specifically designed physical activity intervention and simple physical activity on working memory and motor competence, without comparing the effects of specifically designed physical activity interventions and simple working memory training (i.e., computer-based working memory training) on working memory. A simple working memory training group could be added in future studies to further explore the effects of the physical activity interventions and simple working memory training. Furthermore, our study only considered the influence of two factors (time and group) on the tested variables; other factors, such as gender and socioeconomic status, should be better considered in future research.

## Data availability statement

The original contributions presented in the study are included in the article/Supplementary material. Further inquiries can be directed to the corresponding authors.

## Ethics statement

Ethical review and approval was not required for the study on human participants in accordance with the local legislation and institutional requirements. Written informed consent to participate in this study was provided by the participants' legal guardian/next of kin.

## Author contributions

H-CY and LC designed the experiment and guided the intervention. J-YZ implemented the intervention, collected and analyzed data, and wrote the manuscript. Q-QS, J-MH, and TX implemented the intervention and collected data. D-LW collected data and edited the manuscript. SQ, X-YW, S-BZ, W-WY, S-YH, and C-CL assisted to implement the intervention. All the authors have read and approved of the final version.

## Funding

This study was funded by the Key Program of the National Social Science Foundation (grant number 19ATY010).

## Conflict of interest

The authors declare that the research was conducted in the absence of any commercial or financial relationships that could be construed as a potential conflict of interest.

## Publisher's note

All claims expressed in this article are solely those of the authors and do not necessarily represent those of their affiliated organizations, or those of the publisher, the editors and the reviewers. Any product that may be evaluated in this article, or claim that may be made by its manufacturer, is not guaranteed or endorsed by the publisher.
